# Association between Mediterranean Diet and Development of Multiple Sclerosis: A Systematic Review and Meta‐Analysis

**DOI:** 10.1002/brb3.71205

**Published:** 2026-01-29

**Authors:** Fatemeh Shakouri, Morteza Lotfi, Ali Rostami, Mahnaz Talebi, Sarvin Sanaie, Amirreza Naseri

**Affiliations:** ^1^ Student Research Committee Tabriz University of Medical Sciences Tabriz Iran; ^2^ Neurosciences Research Center (NSRC) Tabriz University of Medical Sciences Tabriz Iran; ^3^ Research Center for Integrative Medicine in Aging, Aging Research Institute Tabriz University of Medical Sciences Tabriz Iran; ^4^ Research Center for Evidence‐Based Medicine, Iranian EBM Centre: A Joanna Briggs Institute (JBI) Center of Excellence Tabriz University of Medical Sciences Tabriz Iran; ^5^ Tabriz USERN Office Universal Scientific Education and Research Network (USERN) Tabriz Iran

**Keywords:** multiple sclerosis, diet, Mediterranean, diet, systematic review, meta‐analysis

## Abstract

**Background:**

Multiple sclerosis (MS) is a chronic inflammatory demyelinating disease of the central nervous system. Given the conflicting evidence regarding the impact of adherence to the Mediterranean diet (MedDiet) on MS development and the lack of a systematic review on this topic, this study aimed to examine this association.

**Methods:**

Following the PRISMA and JBI methods, a search of electronic databases was conducted through PubMed, Embase, Web of Science, and Scopus up to March 2024. Clinical original studies assessing the association between the MedDiet adherence and MS development were included. Risk of bias was evaluated using JBI critical appraisal tools. Meta‐analyses were performed using CMA4 software.

**Results:**

Out of 202 screened records, eight studies, including five case‐control and three cohort studies, met the inclusion criteria. Retrospective evidence from three case‐control studies suggested that MedDiet adherence is associated with decreased odds of MS, whereas cohort studies showed no significant relationship. Based on the meta‐analysis, there was a significant association between high adherence to the MedDiet and reduced odds of MS (fixed‐effect OR: 0.275, 95% CI: 0.11‐0.72, *p*‐value <0.01, least MedDiet adherence as the reference).

**Discussion:**

Although the meta‐analysis showed a significant inverse association between higher adherence to the MedDiet and the odds of MS, the overall body of evidence does not provide strong support for a preventive role of the MedDiet. Considering the limited number of included studies, the predominantly retrospective design, the high risk of bias in several studies, and the substantial observed heterogeneity, further well‐designed prospective studies are needed.

## Graphical abstract

The overall body of evidence does not provide strong support for a preventive role of the Mediterranean diet in multiple sclerosis (components of the graph are retrieved from Servier Medical Art by Servier, licensed under a Creative Commons Attribution 3.0, Unported (CC BY 3.0)).

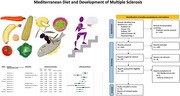

## 1 Introduction

Multiple sclerosis (MS) is an inflammatory demyelinating disease of the central nervous system (CNS). As the most common immune‐mediated disease of the CNS, the pathogenesis of the disease is suggested to be multifactorial, in which both genetic and environmental factors lead to loss of myelin and neuro‐axonal damage caused by T cells, B cells, antibodies, and other immune factors mediated (Sriwastava et al. 2024, Oh et al. 2018), with inflammation and oxidative stress involved (Piñar‐Morales et al. 2025, Daneshvar et al. 2025). Different subtypes of the disease are relapsing‐remitting MS (RRMS), primary‐progressive MS (PPMS), and secondary‐progressive MS (SPMS) (Montalban et al. 2025). MS typically presents with symptoms such as unilateral optic neuritis, partial myelitis, sensory disturbances, or brainstem syndromes like internuclear ophthalmoplegia that develop over several days (McGinley et al. 2021). Most patients experience the first symptoms between 20 and 40 years of age (Marcus 2022, Naseri et al. 2021). The incidence and prevalence of this disease are growing so fast that about one person is diagnosed with MS every five minutes, and about 2.9 million people are affected by MS (Walton et al. 2020, Haki et al. 2024, Boutitah‐Benyaich et al. 2025). Studies show that a healthy diet reduces the risk (Keykhaei et al. 2022), while obesity and a high body mass index (BMI) can increase it (Mohammadi et al. 2024, Aqel et al. 2025), which highlights the importance of developing targeted nutritional guidance and preventive strategies in this regard.

The Mediterranean diet (MedDiet) is a dietary pattern, which is based on a high intake of fruits, vegetables, whole grains, nuts, seeds, and olive oil as the main source of fat; a moderate intake of dairy products, eggs, fish, and wine; deficient intake of red meat and processed food and it is followed by populations around the Mediterranean basin (Guasch‐Ferré and Willett 2021, Obeid et al. 2022). Adherence to MedDiet is suggested to be associated with multiple health effects, including the antioxidant and anti‐inflammatory impacts, lipid‐lowering effects, as well as gut microbiota‐mediated production of metabolites influencing metabolic health (Tosti et al. 2018, Guasch‐Ferré and Willett 2021). Studies indicate that the MedDiet can prevent multiple chronic diseases, including cardiovascular, psychological/neurological, and digestive disorders and cancers (Obeid et al. 2022, Shang et al. 2023). Multiple studies have reported a decline in adherence to the MedDiet over recent decades. For instance, a comparison of Spanish cohorts from 1998 to 2000 and 2019 to 2020 found lower adherence among children and adolescents in the latter cohort (Herrera‐Ramos et al. 2023), which was also evident in other studies, too (Mattavelli et al. 2023, Veronese et al. 2020, León‐Muñoz et al. 2012). The observed decline in adherence over recent years could potentially increase the population risk of the mentioned conditions.

Previously conducted systematic reviews suggested the positive effects of MedDiet in patients with MS. Adherence to MedDiet was found to be associated with reduced fatigue severity and improved quality of life in patients with MS (Abbasi et al. 2025, Abbasi et al. 2023, Abbasi et al. 2023, Kong et al. 2025). Beyond these effects, the MedDiet has been suggested to be associated with a lower risk of MS onset, too (Stoiloudis et al. 2022); however, the potential of MedDiet in the prevention of MS was not addressed in the previous systematic reviews. The proposed protective effects support the theory that the anti‐inflammatory and neuroprotective properties inherent in MedDiet components could play a crucial role in mitigating MS risk (Frye et al. 2024). In addition, modulation of the gut microbiome may also mediate this effect (Rasoulian et al. 2025). This insight invites further investigation into how this dietary pattern influences the development of MS. The evidence related to the effect of the MedDiet and the occurrence of MS is associated with conflicting outcomes (Esposito et al. 2021, Katz Sand et al. 2023, Ertaş Öztürk et al. 2023, Razeghi‐Jahromi et al. 2020); therefore, this study aimed to dissect the evidence regarding this association based on the epidemiological studies.

## 2 Methods

To conduct and report this study, the Joanna Briggs Institute (JBI) Manual for Evidence Synthesis (Aromataris and Munn 2020) and the Preferred Reporting Items for Systematic reviews and Meta‐Analyses (PRISMA 2020) (Page et al. 2021) guidelines were followed.

### 2.1 Eligibility Criteria

All original clinical research publications that discussed the association between adherence to the MedDiet and the odds of MS, without any limitations regarding age, sex, disease severity, or diagnostic criteria, were included, and animal studies, non‐English papers, reviews, letters, editorials, and conference abstracts were excluded.

### 2.2 Data Sources and Search Strategy

A thorough search was conducted in March 2024 across PubMed, Embase, Web of Science, and Scopus to discover the potentially relevant evidence. The following search strategy was used for search in PubMed: (“diet, Mediterranean”[MeSH Terms] OR ((“Mediterranean”[All Fields] OR “Mediterranean”[All Fields]) AND (“diet”[MeSH Terms] OR “diet”[All Fields]))) AND (“Multiple Sclerosis”[MeSH Terms] OR “Multiple Sclerosis”[All Fields]). In addition, the reference lists as well as the citations of each included study and the related studies based on the PubMed database were checked to avoid any overlooked articles, and a monthly alert for new articles was created in this database.

### 2.3 Data Extraction, Selection Process, and Risk of Bias Assessment

The database search results were imported into EndNote 21 software, and after duplicate removal, Rayyan (Ouzzani et al. 2016) was used for the screening process, which was conducted independently by two authors in title/abstract and full‐text stages. Two authors conducted the data extraction, while a third author verified the results. The data extraction table was designed in Microsoft Excel and contained the following items: Identification of the Study; Methodology; Population details; MS diagnostic criteria; MedDiet adherence scale; and the outcomes. Based on the study designs, two authors assessed the risk of bias (RoB) in the included studies utilizing JBI critical appraisal tools (Moola et al. 2020). The details of the critical appraisal tools are presented in Table 2. Any disagreement in screening, data extraction, and/or critical appraisal stages was resolved by discussion or by an expert's opinion.

### 2.4 Quantitative Synthesis

Meta‐analysis was conducted using the CMA4. The level of heterogeneity between the included studies was calculated and reported using the *I* ^2^ index. Studies that reported the odds of MS in tertiles of MedDiet adherence questionnaires were included in the quantitative synthesis, and the final results were presented in a forest plot. The odds ratio was defined as the primary effect size for this study. Considering the small number of included studies in the meta‐analysis, publication bias analysis was not feasible. The level of significance for the *p*‐value was <0.05.

## 3 Results

### 3.1 Study Selection

**FIGURE 1:**
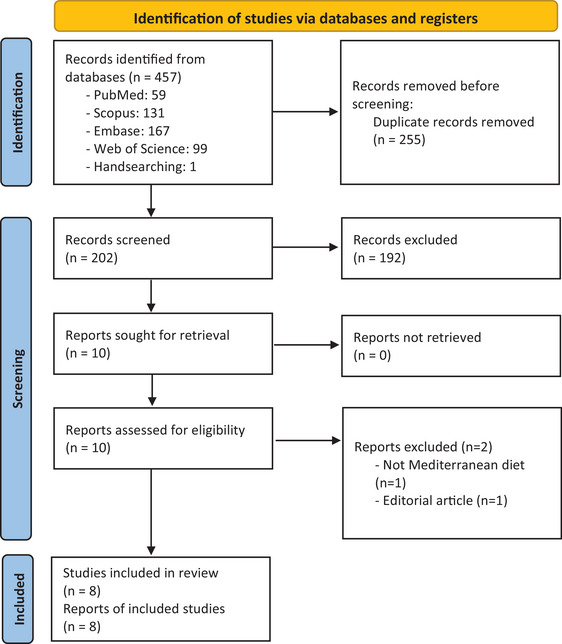
PRISMA flow diagram. *Source*: Page et al. (2021). https://doi.org/10.1136/bmj.n71. For more information, visit: http://www.prisma‐statement.org/.

Of 457 identified records, 202 were screened in the title/abstract stage (Figure 1). Ten records were sought for retrieval and were assessed for eligibility in the full‐text stage. There were two exclusions in this stage; one study did not assess the MedDiet (Materljan et al. 2009), and one study was an editorial article (Cavalla and Vercellino 2023). Finally, eight studies were included (Alfredsson et al. 2023, Black et al. 2019, Barbero Mazzucca et al. 2024, Nitzan et al. 2023, Noormohammadi et al. 2022, Rotstein et al. 2019, Sedaghat et al. 2016, Shang et al. 2023).

### 3.2 Study Characteristics and Results of Individual Studies

In this systematic review, five case‐control (including three population‐based and two hospital‐based studies) and three cohort investigations were included. These studies were conducted in Iran (2), the United Kingdom (2), Australia (1), Sweden (1), Israel (1), and the United States (1). The number of participants ranged from 114,690 to more than 490,000 in the cohort studies and from 100 to 5510 in the case‐control studies. Details of the included studies are presented in Table 1. In addition, the details of the effect size data and adjustments are presented in Supporting Information Material S1.

**TABLE 1:** the characteristics and findings of the included studies.

Study	Design	Setting	Number of participants	Age	Female‐to‐male ratio	MS diagnostic criteria	MedDiet assessment	Results	Conclusion
Black et al. (2019)	Population‐based case‐control	Australian multicenter, Australia	254 cases, 451 controls (matched for age, sex, and study region)	38.6 ± 9.7 cases, 40.0 ± 9.6 Controls	2.96 cases, 3.13 controls	First clinical diagnosis of central nervous system demyelination (FCD) and classic first demyelinating event	Alternate MedDiet score (aMedDiet)	There was no significant association between the aMedDiet score, as well as individual components of the questionnaire, and the risk of FCD.	Adherence to MedDiet is not associated with the risk of MS.
Alfredsson et al. (2023)	Population‐based case‐control	Swedish epidemiological investigation of MS, Sweden	1953 cases, 3557controls (matched for age, sex, and residential area)	Range: 16–70 Age of MS onset: 35.0 ± 10.8	2.35 cases, 2.49 control	2005 and 1010 revisions of McDonald criteria	Self‐reported diet style	After adjustment for ancestry, smoking, alcohol consumption, body mass index, physical activity, and sun exposure habits, MedDiet was associated with a lower risk of MS, even after inclusion of only Nordic origin, restricted to diagnostic gap <2 years, exclusion of subjects with low fish consumption, and exclusion of nondrinkers.	MedDiet may be protective against the risk of subsequently developing MS compared with a Western‐style diet.
Sedaghat et al. (2016)	Hospital‐based case‐control	Major neurological clinics of Tehran, Iran	69 cases, 140 controls (matched for age and sex)	Cases: average: 29, 20–29 (50.7%), 30–39 (31.9%), >40 (17.4%) Control: 20–29 (50.7%), 30–39 (37.9%), >40 (11.4%)	4.75 cases, 4.38 controls	McDonald criteria in the past six months	Level of conformity with MedDiet based on FFQ, one year before diagnosis.	Higher consumption of fruits and vegetables was significantly associated with reduced risk of MS, and higher consumption of refined grains was associated with an increased risk of MS. In both age‐adjusted and multivariate‐adjusted models, the odds of developing MS decreased significantly.	Adherence to MedDiet was associated with reduced risk of MS.
Nitzan et al. (2023)	Population‐based case‐control	Carmel Medical Center, Israel	57 cases, 43 controls (matched for age and sex)	33.6 ± 1.4 cases, 38.1 ± 1.8 controls	2.35 cases, 1.38 controls	2017 version of McDonald criteria for RRMS	A 17‐item MedDiet adherence questionnaire, adapted to the Israeli population	As a secondary outcome of the study, this study found no significant difference between MS patients and healthy controls, regarding the MedDiet score adherence scores and categories.	There is no association between RRMS and MedDiet adherence.
Noormohammadi et al. (2022)	Hospital‐based case‐control	Tehran, Iran	77 cases, 148 controls (matched for sex)	Range: 18—50, 31.0 (28.5–37.0) cases, 35.5 (29.0‐42.8) controls	3.27 cases, 2.7 controls	2017 version of McDonald criteria for RRMS in the last 12 months	Adherence to the MedDiet intervention for neurodegenerative delay	A higher MedDiet score was associated with reduced odds of MS, even after adjustment for age, sex, smoking, total calories, BMI, carbohydrate intake, animal‐based protein intake, and fiber intake. In both basic and adjusted models, MS odds were lower in patients with higher green leafy vegetables, other vegetables, and beans. In the adjusted model, MS was lower in the last tertile of butter and stick margarine. MS was greater in the last tertile of cheese, poultry, pastries, and sweets, and fried/fast foods. Only before adjustment, berry consumption was associated with lower odds of MS.	Higher adherence to MedDiet intervention for neurodegenerative delay may protect against MS.
Rotstein et al. (2019)	Cohort	US nurses’ health study	Over 185,000 (480 incident cases of MS)	30 to 55 and 25 to 42	[only women included]	Self‐reported MS diagnosis, confirmed by contacting the neurologist and medical reports, and supported by MRI evidence in most cases	Alternate MedDiet score (aMedDiet), calculated based on FFQ	aMedDiet (at baseline and mean cumulative) was not statistically significantly associated with the risk of MS, with no meaningful change with omission of BMI at age 18 or addition of socio‐economic status.	There was no evidence for an association between MedDiet and MS risk.
Shang et al. (2023)	Population‐based cohort	UK biobank	114,690 (94 incident cases of MS)	Range: 30–75, 59.0 ± 7.9	1.26	In‐patient records	Alternate MedDiet score (aMedDiet)	After adjustment for age, sex, and total calorie intake (model 1), and also ethnicity, education, income, BMI, smoking, sleep, physical activity, and genetic risk score for longevity, the MedDiet score was not associated with MS risk. None of the components of MedDiet was associated with the risk of MS.	MedDiet is not associated with a lower risk of MS.
Barbero Mazzucca et al. (2024)	Cohort	UK biobank	499,563 (478 incident cases of MS)	Range: 40–69 ≤50: 26.27% (50–60]: 35.26% >60: 28.47%	1.19	In‐patient data	Mediterranean diet (MDS) score, based on FFQ	A nonsignificant trend emerged, suggesting a positive correlation between adherence to the MedDiet and decreased MS risk.	The MedDiet is not significantly associated with MS risk.

Abbreviations: MS: multiple sclerosis; RRMS: relapsing‐remitting MS; MedDiet: Mediterranean diet; FFQ: food frequency questionnaire.

### 3.3 Risk of Bias in Studies

An overview of the results of RoB assessments is presented in Table 2. In the study by Alfredsson et al. (2023), adherence to the MedDiet was investigated using a self‐reported method, and dietary habits 5 years before the disease diagnosis were collected from the cases and controls. In the study by Black et al. (2019), there was RoB regarding the appropriate definition of the cases, which was the first clinical diagnosis of CNS demyelination, which is a common precursor to MS. In addition, the dietary habits in the 12 months before the interview were investigated. In the study by Nitzan et al. (2023), the association between adherence to the MedDiet and MS was investigated as a secondary outcome, so there were no attempts to control for possible confounders. In addition, the duration of adherence to dietary habits is not mentioned. In the study by Noormohammadi et al. (2022), there was a significant difference between the cases and controls regarding age and BMI. Although the utilized questionnaire categorized the duration of dietary habits, the duration of exposure to the MedDiet was unclear. In the study by Sedaghat et al. (2016), dietary habits in the last year were considered. Regarding the cohort studies, there was no significant RoB in the included studies.

**TABLE 2:** Details of the risk of bias assessments.

Study	Design	Q1	Q2	Q3	Q4	Q5	Q6	Q7	Q8	Q9	Q10	Q11
Alfredsson et al. (2023)	Case‐control	Y	Y	Y	N	Y	Y	Y	Y	N	Y	—
Black et al. (2019)	Case‐control	Y	Y	N	Y	Y	Y	Y	Y	N	Y	—
Nitzan et al. (2023)	Case‐control	Y	Y	Y	Y	Y	N	N	Y	N	Y	—
Noormohammadi et al. (2022)	Case‐control	N	N	N	Y	Y	Y	Y	Y	U	Y	—
Sedaghat et al. (2016)	Case‐control	Y	Y	Y	Y	Y	Y	Y	Y	N	Y	—
Rotstein et al. 2019	Cohort	N/A	Y	Y	Y	Y	Y	Y	Y	Y	Y	Y
Shang et al. (2023)	Cohort	U	Y	Y	Y	Y	Y	U	Y	Y	Y	Y
Barbero Mazzucca et al. (2024)	cohort	Y	Y	Y	Y	Y	Y	N	Y	Y	Y	Y

*Note*: JBI checklist for case‐control studies: 1. Were the groups comparable other than for the presence of disease in cases or the absence of disease in controls? 2. Were cases and controls matched appropriately? 3. Were the same criteria used for the identification of cases and controls? 4. Was exposure measured in a standard, valid, and reliable way? 5. Was exposure measured in the same way for cases and controls? 6. Were confounding factors identified? 7. Were strategies to deal with confounding factors stated? 8. Were outcomes assessed in a standard, valid, and reliable way for cases and controls? 9. Was the exposure period of interest long enough to be meaningful? 10. Was an appropriate statistical analysis used?. JBI checklist for cohort studies 1. Were the two groups similar and recruited from the same population? 2. Were the exposures measured similarly to assign people to both the exposed and unexposed groups? 3. Was the exposure measured in a valid and reliable way? 4. Were confounding factors identified? 5. Were strategies to deal with confounding factors stated? 6. Were the groups/participants free of the outcome at the start of the study (or at the moment of exposure)? 7. Were the outcomes measured in a valid and reliable way? 8. Was the follow‐up time reported and sufficient to be long enough for outcomes to occur? 9. Was follow‐up complete, and if not, were the reasons for loss to follow‐up described and explored? 10. Were strategies to address incomplete follow‐up utilized? 11. Was an appropriate statistical analysis used?.

### 3.4 Results of Syntheses

**FIGURE 2:**
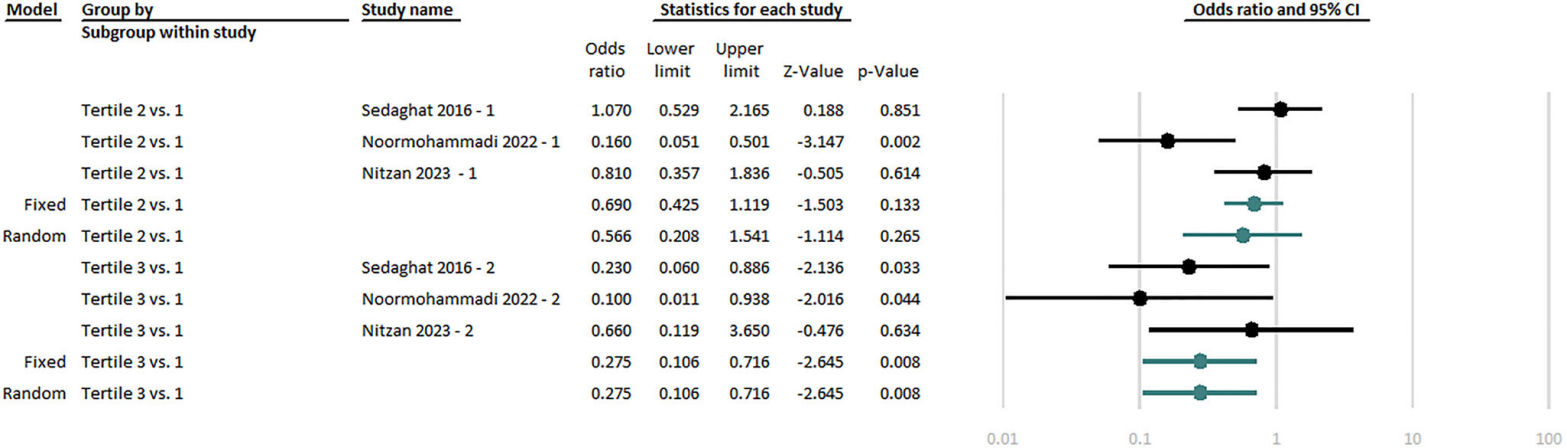
Forest plot of the association between adherence to the Mediterranean diet and the odds of multiple sclerosis.

Three studies categorized the participants’ MedDiet adherence into 3 groups (first group: the least MedDiet adherence, third group: the most MedDiet adherence) and scrutinized the association between the level of adherence and odds of MS (Nitzan et al. 2023, Noormohammadi et al. 2022, Sedaghat et al. 2016). Based on the meta‐analysis, with considering the least MedDiet adherence (tertile 1) as the reference, a moderate adherence to MedDiet was not linked with decreased odds MS (random‐effect OR: 0.57; 95% CI: 0.21–1.54; *p*‐value: 0.26; *I* ^2^: 74.80%; *p*‐value for the heterogeneity: 0.01); however, there was a significant association between high adherence to MedDiet with decreased odds of MS (fixed‐effect OR: 0.27,95% CI: 0.11–0.72, *p*‐value <0.01; *I* ^2^: 00.00%; *p*‐value for the heterogeneity: 0.39) (Figure 2).

## 4 Discussion

This study offered a comprehensive review of the clinical evidence regarding the correlation between MS development and adherence to the MedDiet. Current retrospective evidence in three small case‐control studies (Alfredsson et al. 2023, Noormohammadi et al. 2022, Sedaghat et al. 2016) suggested that higher adherence to the MedDiet and consumption of the essential components, such as fruits, vegetables, legumes, and olive oil, is linked to a lower risk of MS. However, the other case‐control studies (Black et al. 2019, Nitzan et al. 2023), as well as the large cohort studies (Barbero Mazzucca et al. 2024, Rotstein et al. 2019, Shang et al. 2023), were unable to validate this protective correlation. The quantitative synthesis of this study found a decreased odds of MS with a high, but not moderate, adherence to the MedDiet. However, given the limited number of included studies (three), their retrospective design, RoB, and substantial heterogeneity, these findings should be further interpreted cautiously.

The MedDiet's capacity to influence the pathophysiology of chronic conditions through its anti‐inflammatory and antioxidant effects suggests that this dietary style is a potential choice for the prevention of MS (Koelman et al. 2022). Key components of the MedDiet, such as olive oil and grapes, either as fresh fruits or as wine, and citrus fruits, were found to be associated with reduced risk of MS. Gut microbiota is suggested to act as the crucial mediator for this (Cavalla and Vercellino 2023, Conde et al. 2019). Higher adherence to the MedDiet is suggested to be linked with *Bifidobacterium animalis*, a potential probiotic strain, which is proposed to be involved in the disease pathogenesis (Nitzan et al. 2023, Toghi et al. 2019). Higher adherence to the MedDiet is also suggested to be associated with increased 25‐hydroxyvitamin D levels (Barrea et al. 2020), which may decrease the likelihood of MS (Balasooriya et al. 2024). However, the divergent results between case‐control and cohort studies noted in our review emphasize the complexity inherent in nutritional epidemiology and underscore the influence of methodological differences on research findings.

Comprehensive coverage of the evidence and PRISMA‐guided design were the main strengths of this systematic review. The inherent biases associated with case‐control studies, such as recall and selection biases, might have skewed the findings. Additionally, the variability in study duration in cohort studies may explain their contrasting results, as these studies capture a broader range of confounding lifestyle factors over time. The diverse methods used for MedDiet assessment and MS diagnosis across the included studies add another layer of complexity, potentially complicating the interpretation and synthesis of the data. These limitations highlight the challenges in drawing generalized conclusions from heterogeneous study designs.

Despite the preliminary nature of the evidence, promoting the MedDiet may offer public health benefits, and future research should focus on its preventive potential and underlying biological mechanisms in MS. To advance our understanding, future investigations should prioritize the standardization of methodologies for dietary assessment and MS diagnosis. It is crucial to conduct detailed longitudinal studies to elucidate the long‐term effects of the MedDiet on MS risk, including strong measures to control potential confounding factors. Additionally, investigating the molecular mechanism that links dietary patterns to MS development could provide critical insights into the interaction between nutrition and disease. Furthermore, studies exploring the interplay between genetic predispositions and dietary habits could further clarify the conditions under which diet influences the risk of MS. Expanding research to include diverse populations and employing advanced statistical techniques to address heterogeneity in the study designs will be essential for producing more generalizable and conclusive results.

## 5 Conclusion

This systematic review indicates that while some small case‐control studies suggest a potential association between adherence to the MedDiet and reduced odds of MS, the overall evidence remains inconclusive due to high risk of bias and inconsistent findings from cohort studies. Given these limitations, further high‐quality and long‐term cohort studies are essential to clarify the relationship.

## Author Contributions

F.S., M.L., A.R., A.N.: systematic search; study selection, data extraction, risk of bias assessment, preparing the figures, drafting and revising the manuscript. S.S. and M.T.: conceptualization, supervision, and critical editing of the manuscript. All authors approved the final version for submission.

## Funding

The research was supported by Tabriz University of Medical Sciences (grant number: 73644).

## Ethics Statement

The ethics committee of Tabriz University of Medical Sciences reviewed and approved the study protocol (ethics code: IR.TBZMED.REC.1403.707).

## Conflicts of Interest

The authors declare no conflicts of interest.

## Consent for Publication and Consent for Participation

Not applicable.

## Supporting information




**Supplementary Material**: brb371205‐sup‐0001‐SuppMat.xlsx

## Data Availability

All data generated or analyzed during this study are included in this published article and its Supporting Information file.
